# Does Genotype-Specific Phenotype in Patients with Multiple Endocrine Neoplasia Type 2 Occur as Current Guidelines Predict?

**DOI:** 10.3390/cancers16030494

**Published:** 2024-01-24

**Authors:** Teresa Binter, Sabina Baumgartner-Parzer, Marie Helene Schernthaner-Reiter, Melisa Arikan, Lindsay Hargitai, Martin Bruno Niederle, Bruno Niederle, Christian Scheuba, Philipp Riss

**Affiliations:** 1 Division of Visceral Surgery, Department of General Surgery, Medical University of Vienna, 1090 Vienna, Austria; teresa.binter@meduniwien.ac.at (T.B.); melisa.arikan@meduniwien.ac.at (M.A.); lindsay.hargitai@meduniwien.ac.at (L.H.); martin.niederle@meduniwien.ac.at (M.B.N.); bruno.niederle@meduniwien.ac.at (B.N.); christian.scheuba@meduniwien.ac.at (C.S.); 2 Division of Endocrinology and Metabolism, Department of Medicine III, Medical University of Vienna, 1090 Vienna, Austria; sabina.baumgartner-parzer@meduniwien.ac.at (S.B.-P.); marie.schernthaner-reiter@meduniwien.ac.at (M.H.S.-R.)

**Keywords:** multiple endocrine neoplasia type 2, hereditary medullary thyroid carcinoma, c-cell hyperplasia, pheochromocytoma, primary hyperparathyroidism, *RET* mutation, germline mutation

## Abstract

**Simple Summary:**

Multiple endocrine neoplasia type 2 (MEN2) is an autosomal-dominant inherited orphan disease, caused by activating germline mutations of the *RET* gene. This syndrome is mainly characterized by the occurrence of medullary thyroid carcinoma, pheochromocytoma, and primary hyperparathyroidism. The frequency and occurrence of the mentioned manifestations depend on the respective pathogenic variant of the *RET* gene. Recommendations for treatment and follow-up of individuals are currently derived from studies characterizing the genotype-dependent phenotype. In this analysis, we examined the clinical presentation of a cohort of 158 MEN2 patients with different mutations, comparing these findings with the risk profiles of the respective mutation in current guidelines. The results are quite consistent, thereby reinforcing the foundations for optimal clinical management.

**Abstract:**

The clinical manifestation of multiple endocrine neoplasia type 2 (MEN2) in terms of developing medullary thyroid cancer (MTC), pheochromocytoma (PCC), and/or primary hyperparathyroidism (PHPT) is related to the respective pathogenic variant of the *RET* proto-oncogene. The aim of this study is to retrospectively analyze the individual, genotype-dependent clinical manifestations of a large cohort of MEN2 patients. By comparing their clinical profile with currently existing evidence-based knowledge, an optimal therapy and prevention strategy in terms of prophylactic thyroidectomy and clinical follow-up could be ensured. This is a retrospective single-center study of 158 MEN2 patients who were diagnosed and/or surgically treated at a tertiary referral care center between 1990 and 2022. All participants were categorized according to their pathogenic variant of the *RET* proto-oncogene. Subsequently, the clinical manifestation of the disease and its time of occurrence was documented. Our analysis showed results in line with existing studies, except for a considerably lower-than-predicted occurrence of PCC in patients with V804M/L mutations. This study supports the current recommendation regarding the pathogenic variant-dependent management of this rare cancer-associated syndrome.

## 1. Introduction

Multiple endocrine neoplasia type 2 (MEN2) is an autosomal-dominant inherited syndrome caused by an activating germline mutation of the *RET* proto-oncogene (MIM *164761) located on chromosome 10. This syndrome is primarily characterized by the occurrence of medullary thyroid carcinoma (MTC) and pheochromocytoma (PCC), as well as—depending on the clinical subtype—primary hyperparathyroidism (PHPT) [1,2]. MTC is a rare neuroendocrine tumor originating from the parafollicular c-cells of the thyroid gland. This malignant tumor occurs either sporadically or hereditarily within the context of MEN2 syndrome or familial medullary thyroid carcinoma (FMTC), respectively [3]. For patients in an early stage of MTC, the standard curative therapy involves the surgical approach of total thyroidectomy with at least bilateral central neck dissection but in some cases also lateral neck dissection. Regardless of tumor localization, size, and number, tumor-involved lymph nodes evolve early, significantly worsening the prognosis. To cure the disease, an extended lymphadenectomy becomes necessary, if still feasible [4,5,6]. PCC causes the hypersecretion of catecholamines, leading to a wide spectrum of clinical symptoms, some of which can potentially be lethal. However, identifying patients with PCC is challenging, as many individuals present with only a few highly unspecific clinical symptoms. Some even remain asymptomatic. Due to the life-threatening consequences of the manifestation of PCC on the one hand and because of the existing malignant potential of the tumors on the other, early removal is crucial [7]. Due to non-suppressed parathyroid hormone levels, patients with primary hyperparathyroidism (PHPT) exhibit elevated serum calcium levels, leading to the development of renal stones, cortical bone loss, and fractures. This medical condition warrants surgical intervention, mindful of the indications for surgery [8]. MEN2 is an orphan disease with a prevalence of 1–9/100,000 (ORPHA: 653) [9]. The clinical manifestation of the syndrome is related to the position of the respective pathogenic variant of the *RET* proto-oncogene [10,11,12,13,14,15,16,17,18]. More than 100 distinct pathogenic variants of the *RET* proto-oncogene define the variable extent and aggressiveness of MTC, as well as the likelihood of the occurrence of other MEN2-associated diseases [19]. In 95% of cases, patients with classic MEN2A syndrome (MIM #171400) exhibit a *RET* germline mutation in codon 609, 611, 618, or 620 of exon 10, as well as codon 634 in exon 11 [14]. These patients always present with MTC and—depending on the pathogenic variant—also develop PCC and/or PHPT. In the aggressive form MEN2B (MIM #162300), a pathogenic variant in exon 16 (codon M918T) can be detected in 95% of the cases and a mutation in exon 15 (A883F) in less than 5%. About 50% of patients with MEN2B develop PCC [3]. Prophylactic (total) thyroidectomy (TT), as well as screening examinations, should be performed according to the risk classification of the respective pathogenic variant in the 2015 American Thyroid Association (ATA) guidelines (highest/high/moderate risk) [3,19,20]. The aim of this analysis is to review the individual, mutation-dependent clinical manifestations of MEN2 syndrome in a large cohort of patients who were diagnosed, treated, and observed by a tertiary referral care center over the past 32 years, as well as to review whether the genotype-specific phenotype reflects the description in the current guidelines and consensus statements [3,20]. As our department is a center of excellence in endocrine diseases (ENETS center of excellence), the number of treated patients is high. Therefore, a comparison of the study cohort to currently existing evidence-based knowledge could ensure that prevention strategies are consistently optimized, enhancing overall clinical efficacy and outcomes.

## 2. Materials and Methods

This is a retrospective cross-sectional single-center study of 158 patients with MEN2 syndrome who were tested and/or surgically treated at our tertiary care center between 1990 and 2022. All patients who underwent positive genetic testing on exons 5, 8, 10, 11, 13, 14, 15, and 16 for a pathogenic variant of the *RET* proto-oncogene were included. Genetic testing was conducted due to either clinical presentation with MTC or PCC or to familial screening that was suggested and consequently offered to all family members of mutation carriers. DNA was extracted from peripheral blood leucocytes according to standard procedures and subsequent PCR-based sequencing of exons 5, 8, 10, 11, 13, 14, 15, and 16 of the *RET*-proto-oncogene was performed during routine diagnostic procedures as published previously [21]. Patients were identified through a query in the electronic database of the institution of all records created in the above-mentioned period. Those with *RET* germline mutations were identified by manually extracting reports of genetic testing of the archive by authorized persons. Subsequently, the index patients and genetically positive family members were categorized according to their pathogenic *RET* background. Subsequently, all patients meeting the inclusion criteria were manually examined for the occurrence of clinical manifestations of MEN2 syndrome over the whole available observation period. These examinations encompass data regarding performed surgeries, imaging findings, as well as laboratory assessments of calcitonin, CEA, metanephrine concentrations in urine and plasma, calcium, and parathyroid hormone. This analysis describes the disease’s manifestation during follow-up, which ends individually with the age at the time of the last clinical consultation. The clinical manifestation of the disease and its time of occurrence during the observation period are presented by means of descriptive statistics using IBM SPSS Statistics Version 28.0.0.0 (190). Frequencies of categorical variables are reported as percentages, and continuous variables are reported as medians.

## 3. Results

### 3.1. Baseline Characteristics

A total of 222 patients fulfilled the mentioned criteria of a positive genetic testing on exons 5, 8, 10, 11, 13, 14, 15, 16 for a pathogenic variant of the *RET* proto-oncogene. Of these, 64 patients tested positive for variants of unknown significance and were therefore not included in this study, while 158 patients remained to be analyzed regarding genotype-dependent disease manifestation. A total of 129 patients were assigned to the ATA “moderate risk” group, 27 to the ATA “high risk” group, and 2 to the ATA “highest risk” group. The risk groups for the aggressiveness of MTC, defined by the American Thyroid Association, cannot be equated with the defined risk stratifications for the incidence of PCC and PHPT. Thus, different group sizes arose for the other risk groups: the group with an incidence risk of 50%/20–30%/10%/0% for PCC was attributed to 29/44/80/5 out of a total of 158 patients. Patients in the group with an incidence risk of 20–30%/10%/0% for PHPT were attributed to 27/113/18 out of a total of 158 patients. The median (min–max) age of all patients at the time of their last clinical follow-up was 53 (3–91) years. At the time of this analysis, the follow-up can be described in detail as follows: patients in the ATA MTC “moderate risk” category were monitored up to a median (min–max) age of 55 (5–91), patients in the “high risk” category were monitored up to a median (min–max) age of 43 (3–72), and the 2 patients in the “highest risk” category were at the ages of 8 and 46 years at their last follow-up.

### 3.2. Genotype–Phenotype Correlation: Medullary Thyroid Carcinoma

ATA “highest risk”: two of two patients with a pathogenic variant in codon M918T underwent total thyroidectomy (TT). Both presented with MTC (N0|N1|NX: 0|2|0). Median (min–max) age at TT was 15.5 (8–23) years.

ATA “high risk”: 26 of 27 [96.3%] patients with a C634F/G/R/S/W/Y mutation underwent TT. Twenty-four of twenty-seven [88.9%] presented with MTC (N0|N1|NX: 12|7|5). Median (min–max) age at TT with the result of MTC was 24.5 (4–72) years.

**Figure 1:**
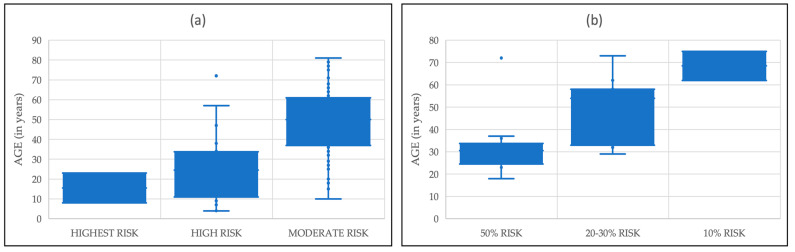
Age at diagnosis of (**a**) MTC in relation to ATA risk classification; (**b**) PCC in relation to ATA incidence risk.

ATA “moderate risk”: 106 of 129 [82.2%] patients within this category underwent TT. Of the 106, 11 [10.4%] presented with neoplastic c-cell hyperplasia (nCCH) and 83 of 106 [78.3%] presented with MTC (N0|N1|NX: 52|27|4). The median (min–max) age at TT with the result of nCCH was 27 (5–46) and the median (min–max) age at TT with the result of MTC was 50 (10–81) years. A more detailed breakdown of the clinical manifestation of individual mutations can be found in Figure 1 and Table 1.

### 3.3. Genotype–Phenotype Correlation: Pheochromocytoma

ATA “50%-incidence”: one of two patients with a codon M918T mutation presented with PCC at the age of 31. Fifteen of twenty-seven [55.6%] patients with a codon C634F/G/R/S/W/Y mutation presented with PCC. Median [min–max] age at adrenalectomy (AD) was 29 (18–72) years.

ATA “20–30%-incidence”: seven of twenty-five [28.0%] patients with a codon C611F/G/S/Y/W mutation presented with PCC. Median [min–max] age at AD was 55 (29–86) years. Two of ten [20.0%] patients with a codon C618F/R/S/Y mutation presented with PCC. Their median (min–max) age at AD was 47.5 (37–58) years. Two of eight [25.0%] patients with a codon 620F/R/S/Y mutation presented with PCC. Median (min–max) age at AD was 43 (32–54) years. One carrier of a C630R mutation had not yet presented with PCC until the end of the follow-up at the age of 61 years.

ATA “10% incidence”: one of nine [11.1%] patients with a codon L790F mutation presented with PCC at the age of 62 years. No patient with a codon G533C mutation presented with PCC. Of 56 patients with a codon V804M/L mutation, none presented with PCC until a median (min–max) age of 57 (7–88) years at the time of their last follow-up examination. One of thirteen [7.7%] patients with a codon S891A mutation presented with PCC at the age of 75 years.

ATA “0%-incidence”: Accordingly, 0 of 5 patients with a codon E768D mutation presented with PCC.

A more detailed breakdown can be found in Figure 1 and Table 2.

### 3.4. Genotype–Phenotype Correlation: Primary Hyperparathyroidism

ATA “20–30%-incidence”: seven of twenty-seven [25.9%] patients with a codon C634F/G/R/S/W/Y mutation presented with PHPT. Median (min–max) age at parathyroidectomy (PT) was 33 (14–72) years. 

ATA “10%-incidence”: one of twenty-five [4.0%] patients with a codon C611F/G/S/Y/W mutation presented with PHPT. The age at PT was 23 years. The only patient with a C630R mutation presented with PHPT and underwent PT at the age of 58 years. Of 56 [7.1%] patients with a codon V804L/M mutation, 4 presented with PHPT undergoing PT at a median (min–max) age of 59.5 (30–79) years. In the group with a mutation in codon C618F/R/S/Y, C620 F/R/S/Y or S891A, nobody presented with PHTH. 

ATA “0%-incidence”: We investigated 18 patients whose mutations belong to the group without PHPT risk (G533C, E768D, L790F, M918T). Accordingly, no patient presented with PHPT in this group. 

A detailed breakdown can be found in Table 3.

## 4. Discussion

### 4.1. Genotype-Related Occurrence and Aggressiveness of MTC

According to the revised American Thyroid Association Guidelines of 2015 [3], the MTC “highest risk” (ATA-HST) and “high risk” (ATA-H) groups show an almost 100% MTC incidence at a young age. In the group with „moderate risk” (ATA-MOD), gene carriers present with MTC at a comparatively older age. The classification of MTC is based on aggressiveness in terms of early onset. MTC occurrence per se is 100% according to guidelines and studies [14]. Based on this knowledge, prophylactic thyroidectomy is recommended in children in the first year or even month of life in ATA-HST, before 5 years of age based on serum calcitonin levels in ATA-H, and when serum calcitonin levels become elevated in “ATA MOD” [3,17]. In adults, annual testing is recommended for normal serum calcitonin values and thyroidectomy with or without neck dissection immediately when calcitonin levels start rising. In this study, almost all patients in the ATA-HST and ATA-H risk categories presented with MTC but in the ATA-MOD group, only 64.3% developed MTC at the time of diagnosis of this hereditary disease. As Niederle et al. already demonstrated, the postponement of prophylactic TT is justified, but only in consideration of certain co-factors. Thus, the genotype–age–calcitonin concept (GAC-concept) was described to determine the timing and extent of surgery in patients with moderate-risk mutations [20]. Only 3 of 92 patients (all associated with the „moderate risk” group) presented with histologic findings without a desmoplastic stromal reaction (DSR). DSR is considered a reliable parameter for the lymph node metastatic potential of sporadic MTC [4,22]. Niederle et al. already described a frequent occurrence of DSR in patients with various mutations of the *RET* proto-oncogene in an analysis in 2021 [4]. 

### 4.2. Genotype-Related Incidence of PCC

The frequency of PCC, as well as its synchronous or metachronous occurrence with MTC/nCCH, largely corresponds to the known risk predictions [3,23]. Some conspicuities emerge: in the group with a mutation in codon V804M/L, there was not a single PCC case among 56 patients despite a 10% probability. In MEN2A patients with a mutation in codon V804M/L, the occurrence of PCC has been reported in some studies, but also only in very rare cases, sometimes not at all [24,25,26,27,28]. Nevertheless, the very uncomplicated screening for PCC is probably not negligible. Considering the regular MTC screening/follow-up examinations that are required anyway, PCC screening represents little additional effort for those affected.

### 4.3. Genotype-Related Incidence of PHPT

When it comes to genotype-specific manifestations of PHPT, the current analysis showed results in line with the mutation-dependent risk groups of PHPT occurrence in the ATA guidelines (0%, 10%, and 20–30%, respectively) [3,29,30]. In our entire MEN2 cohort—regardless of individual mutations—an 8% prevalence of PHPT was observed. This corresponds exactly to the result of a previous analysis conducted by Holm et al., in which more than 200 MEN2 patients were thoroughly examined regarding the occurrence of PHPT and a lower frequency than that reported in previous studies (19–35%) was indicated [31,32,33,34,35]. The result of 8% also aligns with recent findings by Machens et al., in which a penetrance of 10.8% for PHPT was observed in MEN2 patients. The use of more stringent definition criteria for PHPT over the past years has been pointed out as a possible cause of its reduced incidence [36].

### 4.4. Limitations

In addition to the known limitations of a retrospective study regarding data quality and retrospective biases, it must be considered that no lifelong follow-up is available for all patients. Therefore, a complete understanding of the true penetrance of certain disease manifestations over an entire lifespan of every individual cannot be guaranteed. The findings must be interpreted within the context of these limitations, highlighting the importance of prospective studies with extended follow-up periods to elucidate the lifelong course of MEN2 syndrome and its associated clinical manifestations more accurately. Furthermore, the value of this analysis is limited due to the small number of cases of this orphan disease. 

## 5. Conclusions

This examination of the genotype–phenotype correlation in a substantial cohort of individuals carrying a pathogenic variant of the RET proto-oncogene with the potential to initiate the manifestation of multiple endocrine neoplasia syndrome aligns with existing guidelines. Almost all patients in this study were classified under the ATA-HST and ATA-H risk categories and presented with MTC. However, within the ATA-MOD group, only two-thirds manifested MTC at the time of this analysis. Postponing prophylactic total thyroidectomy is justified but only in consideration of genotype, age, and calcitonin levels. The occurrence of PCC in this cohort largely corresponds to the risk predictions outlined in the guidelines. Regarding PHPT, we observed a tendency towards a lower incidence, as already reported in other recent studies, which may be associated with the application of stricter definition criteria for diagnosing PHPT. Consequently, the management of patients with multiple endocrine neoplasia type 2 appears reasonable.

## Figures and Tables

**Table 1:** Genotype-related occurrence of MTC in MEN2.

ATA RISK MTC	MUTATION	CARRIERS	nCCH	MTC	MTC pN0	MTC pN1	MTC pNx	AGE At MTC
HST	M918T	2	-	2 (100)	-	2 (100)	-	15.5 (8–23)
HIGH	C634F/G/R/S/W/Y	27	-	24 (88.9)	12 (50.0)	7 (29.2)	5	24.5 (4–72)
	A883F	-	-	-	-	-	-	-
MOD	G533C	2	-	1 (50.0)	-	1 (100.0)	-	29
	C609F/G/R/S/Y	-	-	-	-	-	-	-
	C611F/G/S/Y/W	25	6 (24.0)	13 (52.0)	6 (46.2)	7 (53.8)	-	50.5 (34–79)
	C618F/R/S/Y	10	-	9 (90.0)	7 (77.8)	2 (22.2)	-	38 (27–59)
	C620F/R/S/Y	8	1 (12.5)	7 (87.5)	4 (57.1)	2 (57.1)	1	36 (21–55)
	C630R/Y	1	-	1 (100)	1 (100)	0	-	58
	D631Y	-	-	-	-	-	-	-
	K666E	-	-	-	-	-	-	-
	E768D	5	-	4 (80.0)	3 (75.0)	1 (75.0)	-	48 (39–68)
	L790F	9	-	7 (77.8)	4 (57.1)	2 (28.6)	1	55 (10–81)
	V804M/L	56	4 (7.1)	29 (51.8)	18 (62.1)	9 (31.0)	2	56 (16–77)
	S891A	13	-	12 (92.3)	9 (75.0)	3 (25.0)	-	50 (15–75)
	R912P	-	-	-	-	-	-	-

MTC: number [percentage] of patients with medullary thyroid carcinoma; nCCH: number [percentage] of patients without MTC but with neoplastic c-cell hyperplasia; HST: ATA highest risk for MTC; HIGH: ATA high risk for MTC; MOD: ATA moderate risk for MTC; age at MTC: median [minimum–maximum] age at time of total thyroidectomy with diagnosis of MTC.

**Table 2:** Genotype-related occurrence of PCC in MEN2.

ATA INCIDENCE PCC	MUTATION	CARRIERS	PCC	UNILATERAL	BILATERAL	AGE AT PCC
50%	D631Y	-	-	-	-	-
	C634F/G/R/S/W/Y	27	15 (55.6)	4	11	29 (18–72)
	A883F	-	-	-	-	-
	M918T	2	1 (50.0)	-	1	31
20–30%	C609F/G/R/S/Y	-	-	-	-	-
	C611F/G/S/Y/W	25	7 (28.0)	5	2	55 (29–86)
	C618F/R/S/Y	10	2 (20.0)	-	2	47.5 (37–58)
	C620F/R/S/Y	8	2 (25.0)	2	-	43 (32–54)
	C630R/Y	1	-	-	-	-
10%	G533C	2	-	-	-	-
	K666E	-	-	-	-	-
	L790F	9	1 (11.1)	1	-	62
	V804M/L	56	-	-	-	-
	S891A	13	1 (7.7)	1	-	75
0%	E768D	5	-	-	-	-
	R912P	-	-	-	-	-

PCC: number [percentage] of patients with pheochromocytoma; age at PCC: median [minimum–maximum] age at time of adrenalectomy with result of pheochromocytoma.

**Table 3:** Genotype-related occurrence of PHPT in MEN2.

ATA INCIDENCE PHPT	MUTATION	CARRIERS	PHPT	AGE AT PHPT
20–30%	C634F/G/R/S/W/Y	27	7 (25.9)	33 (14–72)
10%	C609F/G/R/S/Y	-	-	-
	C611F/G/S/Y/W	25	1 (4.0)	23
	C618F/R/S/Y	10	-	-
	C620F/R/S/Y	8	-	-
	C630R/Y	1	1 (100)	58
	V804M/L	56	4 (7.1)	59.5 (30–79)
	S891A	13	-	-
0%	G533C	2	-	-
	D631Y	-	-	-
	K666E	-	-	-
	E768D	5	-	-
	L790F	9	-	-
	A883F	-	-	-
	R912P	-	-	-
	M918T	2	-	-

PHPT: number [percentage] of patients with primary hyperparathyroidism; age at PHPT: median [minimum-maximum] age at time of parathyroidectomy.

## Data Availability

We are committed to confidentiality and ethical integrity of the information collected in this study. Upon reasonable request, the corresponding author will provide access to the data collected for this study.
